# Skin microbiota differs drastically between co-occurring frogs and newts

**DOI:** 10.1098/rsos.170107

**Published:** 2017-04-05

**Authors:** Molly C. Bletz, R. G. Bina Perl, Miguel Vences

**Affiliations:** Zoologisches Institut, Technische Universität Braunschweig, Mendelssohnstr. 4, 38106 Braunschweig, Germany

**Keywords:** 16S amplicon sequencing, Amphibia, cutaneous bacterial communities, frogs, newts

## Abstract

Diverse microbial assemblages inhabit amphibian skin and are known to differ among species; however, few studies have analysed these differences in systems that minimize confounding factors, such as season, location or host ecology. We used high-throughput amplicon sequencing to compare cutaneous microbiotas among two ranid frogs (*Rana dalmatina, R. temporaria*) and four salamandrid newts (*Ichthyosaura alpestris, Lissotriton helveticus, L. vulgaris, Triturus cristatus*) breeding simultaneously in two ponds near Braunschweig, Germany. We found that bacterial communities differed strongly and consistently between these two distinct amphibian clades. While frogs and newts had similar cutaneous bacterial richness, their bacterial composition strongly differed. Average Jaccard distances between frogs and newts were over 0.5, while between species within these groups distances were only 0.387 and 0.407 for frogs and newts, respectively. At the operational taxonomic unit (OTU) level, 31 taxa exhibited significantly different relative abundances between frogs and newts. This finding suggests that chemical or physical characteristics of these amphibians' mucosal environments provide highly selective conditions for bacterial colonizers. Multi-omics analyses of hosts and their microbiota as well as directed efforts to understand chemical differences in the mucosal environments (e.g. pH), and the specificities of host-produced compounds against potential colonizers will help to better understand this intriguing pattern.

## 1. Introduction

The mucosal environment of amphibian skin provides a habitat in which microbes can live. These microbial assemblages form communities that interact and can provide functional benefits for the host, including protection against skin pathogens, such as *Batrachochytrium dendrobatidis* (*Bd*) [1]. Numerous studies have examined these benefits in detail and have shown how symbiotic microorganisms can inhibit pathogens and in turn promote host immunity [2–4].

Initial assembly of these communities is potentially derived from vertical, horizontal and environmental transmission [5–9]. While vertical transmission may provide initial colonizers for some amphibian species [9], maintenance of amphibian skin microbial communities probably results from continual exchange with the environment [5]. However, amphibian skin microbiotas typically do not represent random assemblages of environmental bacteria [10,11], and these cutaneous communities have been found to differ between hosts and across populations of the same host [11–14]. Comparisons among species are often confounded by comparisons among individuals collected at different points in time, different locations, different life-history phases (adults versus larvae, or aquatic versus terrestrial), or by comparing species of largely different ecology (e.g. ground-dwelling versus arboreal). The existence of interspecific differences is clear from several studies, e.g. from the comparisons of captive individuals living for several generations under fully comparable conditions [14]. However, field studies have often compared species from different genera; it remains unexplored whether cutaneous microbial composition or diversity consistently differs to the same extent when comparing species from within major amphibian clades or genera as when comparing species from between these major clades (i.e. does host phylogeny play a role in amphibian microbial differences?).

The physical and chemical structure of the skin ecosystem differs between amphibian species, and these differences probably play a role in shaping their cutaneous microbiota. For instance, amphibian species differ in the composition of host-produced mucins, glycoproteins, antimicrobial peptides, and synthesized or sequestered alkaloids within the mucosal environment [15–18]. These compounds can influence the microbial taxa able to colonize and therefore probably dictate microbial diversity and community structure within this environment. Although the bacterial communities of amphibians appear to be assembled from the environment and—at least for the majority of bacteria—probably are not derived from vertical transmission, some differences among major amphibian clades can be hypothesized to exist. Considering that the major extant amphibian clades are as ancient as the Palaeozoic [19,20], the presence of clade-specific characteristics of the immune system (e.g. antimicrobial peptides) or skin morphology can be hypothesized, and these could act as environmental filters influencing colonization by different bacterial taxa. Sampling amphibians within their habitats can allow us to describe their natural cutaneous microbial communities and explore their ecological assembly patterns, i.e. host-filtering, or neutral processes.

Here, we study members of the amphibian community inhabiting two ponds in the Braunschweig area, Germany, as a model system to understand differences among cutaneous bacterial communities in ancient amphibian clades. We compare the microbiotas of two representatives of the anuran family, Ranidae, with those of four representatives of the caudate family, Salamandridae. The focal species co-occur in the ponds and share numerous aspects of their breeding phenology. They spend most of the year in their terrestrial phase, migrate in early spring to their breeding pond, and undergo skin morphological transformations between the terrestrial and aquatic phase. We sampled the six species in the same time period to minimize seasonal effects, specifically addressing the following two questions: (i) do frogs and newts, i.e. representatives of two amphibian clades evolving separately since the Palaeozoic, differ in their bacterial communities, and (ii) do species within these amphibian groups (i.e. species within the families Ranidae and Salamandridae) share more bacterial taxa with each other than with species of the other group?

## 2. Methods

### 2.1. Sample collection

Between 17 and 20 March, we collected skin microbial samples from two distinct locations near Braunschweig, Lower Saxony, Germany: Elm (latitude 52.213, longitude 10.830) and Kleiwiesen (52.328, 10.582). The Elm is a protected nature park on Triassic limestone with the largest beech forest in northern Germany (length: approx. 25 km, width: 3–8 km). The samples herein were taken near Königslutter-Lelm at the northeastern part of the forested range, in a pond completely surrounded by mixed deciduous and pine forest. The area of the Kleiwiesen (approx. 6 km^2^) is a nature sanctuary located north of Braunschweig. The area harbours multiple artificially created ponds and its fertile soil (marine clay) promotes the growth of a variety of different vegetation, including grass, shrubs and trees. The ponds are surrounded by deciduous forest and by meadows. Both sampling sites are located at a (linear) distance of 21.4 km from each other and differ in elevation (Kleiwiesen: 92 m, Elm: 238 m above sea level). We sampled two frog species, *Rana dalmatina* (Elm), and *R. temporaria* (Elm and Kleiwiesen), as well as four newt species, *Ichthyosaura alpestris* (Elm and Kleiwiesen)*, Lissotriton helveticus* (Elm), *L. vulgaris* (Elm and Kleiwiesen) and *Triturus cristatus* (Elm and Kleiwiesen). In the two ponds, the focal amphibian species co-occur in exactly the same aquatic microhabitat, and thus in their aquatic phase are exposed to the same environmental reservoir of bacteria. All of the species are morphologically and genetically distinct, with rare events of hybridization recorded only among the two species of *Lissotriton* [21]. All six species occur at Elm (*L. helveticus* being very rare), while *R. dalmatina* and *L. helveticus* are absent from Kleiwiesen. Research and sample collection permits were obtained from the conservation authorities of Helmstedt and Braunschweig.

Amphibians were captured either directly by gloved hands (clean nitrile gloves were used for each individual) or dip nets. Each captured individual was held with unique gloves, rinsed with 50 ml of filtered (0.22 µm) deionized water to remove debris and transient microbes, and swabbed on its ventral surface 10 times (1 time = an up and back stroke) using a sterile MW113 swab (Medical Wire and Equipment, Corsham, UK). Swabs were stored in unique sterile vials and transferred into a −20°C freezer within 2 h after collection. Amphibians were returned to the ponds immediately after sampling of all individuals was complete at a given site. Sampling resulted in 18 samples from *R. dalmatina* (Elm, *n* = 18; Kleiwiesen *n* = 0), 40 samples from *R. temporaria* (Elm, *n* = 11; Kleiwiesen, *n* = 29), 18 samples from *I. alpestris* (Elm, *n* = 16; Kleiwiesen, *n* = 2), eight samples from *L. helveticus* (Elm, *n* = 8; Kleiwiesen, *n* = 0), 41 samples from *L. vulgaris* (Elm, *n* = 14; Kleiwiesen, *n* = 27) and 16 samples from *T. cristatus* (Elm, *n* = 10; Kleiwiesen *n* = 6). All newt samples were obtained from aquatic-phase adults, and all frog samples were taken from adults in their breeding period when they lead an almost fully aquatic life for a short time. Uneven sampling is explained by our effort to sample all six species in exactly the same time period during which only a limited number of individuals of some species could be found. Aquatic microbial community samples were also taken from the pond water at the time of amphibian sampling from both locations.

### 2.2. DNA extraction and sequencing

Whole-community DNA was extracted from swabs with the MoBio PowerSoil-htp 96-well DNA isolation kit (MoBio, Carlsbad, CA, USA) following the manufacturer's protocol with minor adjustments, i.e. a 10-minute incubation at 65°C after C1 addition, increased centrifuge time to account for slower rotor speed available and 10-minute incubation at room temperature after C6 addition to the spin column. DNA extracts were stored at −20°C until further processing. The dual-index approach was used to PCR-amplify the V4 region of the bacterial 16S rRNA gene using the 515F and 806R primers [22]. Both the forward and reverse primers included the Illumina adapter and a unique barcode tag. Polymerase chain reaction (PCR) methods followed those used in a previous study [23]. Samples were pooled together in approximately equal concentration (as determined by gel band strength), and then cleaned using a Qiagen MiniElute Gel Extraction Kit. A Qubit fluorometer with a broad-range dsDNA kit was used to quantify the DNA after clean-up. The pooled PCR amplicons of all samples were sequenced using paired-end 2 × 250 v2 chemistry on an Illumina MiSeq sequencer at the Helmholtz Center for Infection Biology in Braunschweig, Germany.

### 2.3. Sequence processing

Quantitative Insights Into Microbial Ecology (MacQIIME v. 1.9.1) was used to process all sequence data, unless otherwise stated [24]. Forward and reverse reads from each sample were joined using Fastq-join with default settings [25]. Joined reads were quality-filtered to remove low-quality sequences with the QIIME defaults. Sequences were also filtered by read length to include only the reads with a length between 250 and 253 bp (usegalaxy.org). Chimeras were identified on a per-sample basis using usearch61 denovo-based detection within QIIME (http://drive5.com/usearch/usearch_docs.html) [26], and subsequently removed. After filtering, 644 334 sequences remained for analysis. Sequences were clustered into operational taxonomic units (OTUs) at 97% similarity using an open reference OTU-picking strategy ([27], http://qiime.org/tutorials/open_reference_illumina_processing.html). Sequences were first matched to the SILVA 119 (24 July 2014) release (https://www.arb-silva.de) and non-matching reads were subsequently clustered de novo using UCLUST [28]. The most abundant sequence from each OTU was selected as a representative sequence. These representative sequences were aligned using PyNAST [29] and taxonomy was assigned using the RDP classifier [30] with the SILVA 119 taxonomy as the reference. A phylogenetic tree was built using FastTree [31]. OTUs with less than 0.001% of the total reads were removed [32]. All samples were rarefied at 1000 reads to allow inclusion of a large portion of the samples and capture the majority of the diversity present within these skin bacterial communities.

### 2.4. Statistical analysis

Species richness and diversity was estimated with the following metrics: OTU richness, Chao1, Shannon and Faith's phylogenetic diversity. ANOVAs were performed in R (3.2.1) to compare diversity among species, amphibian groups and locations. More specifically, two 2-factor ANOVAs were performed: (i) with species and location, and (ii) with amphibian order and location.

Beta diversity was calculated with the Weighted Unifrac, Unweighted Unifrac and the Bray–Curtis metric, and visualized with Principal Coordinates Analysis (PCoA). Differences in beta diversity among amphibian hosts and locations were tested with a Permutational Multivariate Analysis of Variance (PERMANOVA) using Primer7 [33].

To determine the OTUs driving the observed differences between frog and newt bacterial communities, the linear discriminant analysis (LDA) effect size (LEfSe) method was used [34] on an OTU table modified to include only OTUs found on all individuals of at least one amphibian species. This filtering was completed to (i) identify OTUs that were prevalent and abundant and therefore more likely to be true autochthonous members of the skin bacterial community, and (ii) to reduce the number of comparisons being performed. Amphibian order (frog or newt) was set as the class variable and location (Elm or Kleiwiesen) was set as the subclass variable. The default parameters, including all-against-all multi-class analysis, *α* = 0.05, and least discriminant score (LDA) more than 2, were used. LEfSe analyses were completed on the Galaxy Web platform. The representative sequences of each of the differentially abundant OTUs were used in BLAST searches against the full NCBI nucleotide database as of February 2017 to explore in what other environments, if any, these OTUs have been found.

Core microbiomes were calculated and were defined as those OTUs present on 90% of individuals of a respective species at a given location.

As a measure for comparing the strength of the observed differences, we calculated average pair-wise Jaccard distances between amphibian species from their respective locations. These distances were calculated based on an OTU table containing OTUs present on at least 90% of individuals of at least one amphibian species (i.e. the combined Core-90 OTUs). Jaccard distances were used in order to evaluate differences based on a matrix derived from presence–absence-based data, and the filtering excluded rare OTUs.

## 3. Results

**Figure 1.**
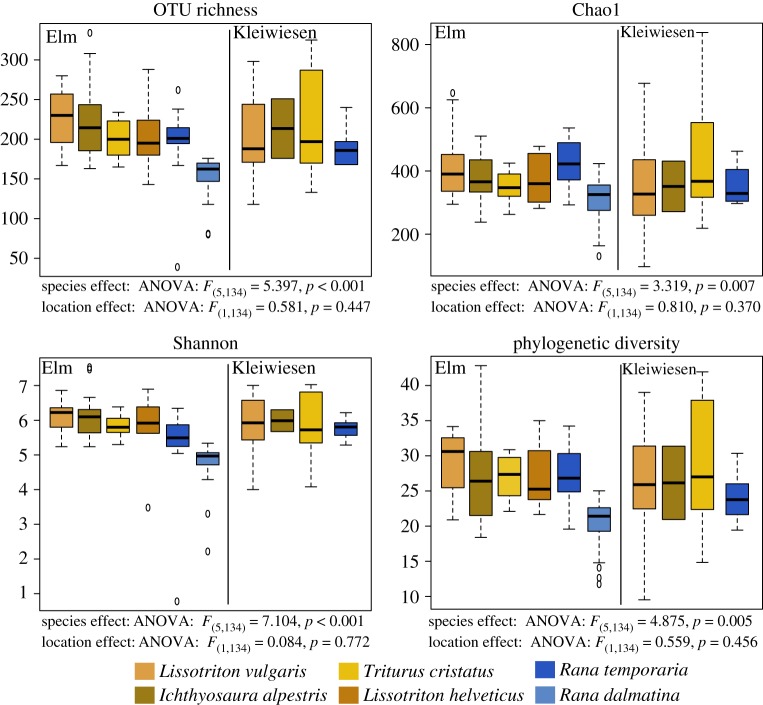
Richness and diversity of skin microbial communities across species at the two sampling sites, Elm and Kleiwiesen (both close to Braunschweig, Germany), using four metrics: OTU richness, Chao1, Shannon diversity and Faith's phylogenetic diversity. Amphibian species are presented with the species sampled at both sites first, followed by those only sampled at one location within each amphibian order and site. Main effect ANOVA results for species and location are provided below each plot.

Species richness and phylogenetic diversity differed across amphibian species and not between locations (figure 1). This species effect was driven by *R. dalmatina* exhibiting lower richness and diversity than the other species (table 1). There was no main effect of amphibian order for Chao1, OTU richness or phylogenetic diversity; however, there was an effect for Shannon diversity (ANOVA, *F*_1,138_ = 9.479, *p* = 0.002). Newts harboured a greater diversity with respect to the Shannon Index.

**Table 1.** Significant pair-wise *post hoc* comparisons for species richness and diversity indices. n.s., not significant.

comparison	Shannon	phylogenetic diversity	OTU richness	Chao1
*R. dalmatina–I. alpestris*	*p* < 0.001	*p* = 0.007	*p* < 0.001	n.s.
*R. dalmatina–L. vulgaris*	*p* < 0.001	*p* = 0.001	*p* < 0.001	n.s.
*R. dalmatina–L. helveticus*	*p* = 0.01	n.s.	n.s.	n.s.
*R. dalmatina–T. cristatus*	*p* = 0.0046	n.s.	n.s.	n.s.
*R. dalmatina–R. temporaria*	*p* < 0.001	*p* < 0.001	*p* < 0.001	*p* < 0.001

**Figure 2.**
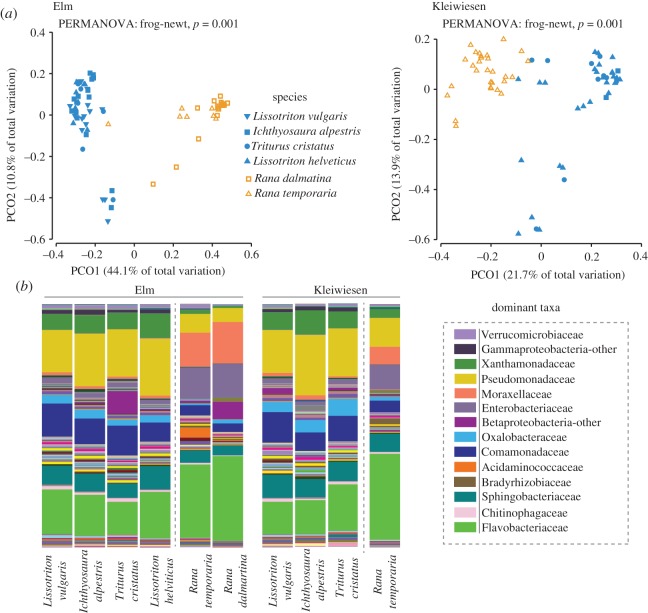
Host-associated cutaneous bacterial communities of frog and newts. (*a*) Principal coordinates analysis of Bray–Curtis beta diversity matrices for amphibians at Elm and Kleiwiesen. PERMANOVA results are provided. (*b*) Relative abundances of bacterial taxa at the family level for hosts at the two sampling locations, Elm and Kleiwiesen (close to Braunschweig, Germany). Taxonomic information for dominant groups is listed at the side. For the bar plots, amphibian species are presented with the species sampled at both sites first, followed by those only sampled at one location within each amphibian order and site.

The cutaneous bacterial community structure differed between frog and newt hosts at both Elm and Kleiwiesen (figure 2, PERMANOVA Pseudo-*F* = 31.072 (Elm), 8.4498 (Kleiwiesen), *p* (MC) = 0.001), while no significant differences were observed between species within their respective amphibian orders (figure 2, PERMANOVA, Pseudo-*F* = 1.327 (Elm), 0.8123 (Kleiwiesen), *p* (MC) > 0.05). The average Jaccard distance between samples of amphibian groups was 0.546 ± 0.067 (s.d.) and 0.528 ± 0.069 (s.d.) for Elm and Kleiwiesen, respectively. Within each amphibian group, the average Jaccard distance across both localities was 0.407 ± 0.095 (s.d.) and 0.387 ± 0.07 (s.d.) among newt samples and frog samples, respectively. More closely related newt species (i.e. those from the same genus) were not more similar to each other than to other species from different genera. Pair-wise distances between species are provided in table 2.

**Table 2.** Pair-wise Jaccard distances between species for Elm and Kleiwiesen.

	Elm	Kleiwiesen
comparison	average distance (±s.d.)	average distance (±s.d.)
*Rana dalmatina<>Ichthyosaura alpestris*	0.558 ± 0.056	—
*Rana dalmatina<>Lissotriton helveticus*	0.567 ± 0.054	—
*Rana dalmatina<>Lissotriton vulgaris*	0.573 ± 0.073	—
*Rana dalmatina<>Triturus cristatus*	0.580 ± 0.076	—
*Rana temporaria<>Ichthyosaura alpestris*	0.519 ± 0.056	0.554 ± 0.044
*Rana temporaria<>Lissotriton helveticus*	0.525 ± 0.051	—
*Rana temporaria<>Lissotriton vulgaris*	0.536 ± 0.072	0.521 ± 0.095
*Rana temporaria<>Triturus cristatus*	0.544 ± 0.080	0.510 ± 0.070
*Ichthyosaura alpestris<>Lissotriton helveticus*	0.377 ± 0.079	—
*Ichthyosaura alpestris<>Lissotriton vulgaris*	0.394 ± 0.084	0.403 ± 0.148
*Ichthyosaura alpestris<>Triturus cristatus*	0.407 ± 0.105	0.422 ± 0.096
*Triturus cristatus<>Lissotriton helveticus*	0.402 ± 0.111	—
*Triturus cristatus<>Lissotriton vulgaris*	0.420 ± 0.107	0.451 ± 0.133
*Lissotriton helveticus<>Lissotriton vulgaris*	0.390 ± 0.086	—
*Rana dalmatina<>Rana temporaria*	0.388 ± 0.071	—

Frog and newt bacterial communities were composed of many of the same families, but the relative abundances of these taxa differed (figure 2*b*). Flavobacteriaceae (Frog/Newt: 33.4/15.8%), Enterobacteriaceae (12.3/1.7%) and Moraxellaceae (12.7/1.2%) exhibited greater relative abundance on frogs, while Pseudomonadaceae (8.4/20.7%), Comamonadaceae (4.2/10.8%), Xanthomonadaceae (2.0/7.9%) and Oxalobacteraceae (1.2/3.6%) were more abundant on newts. The pond water was composed of the following dominant groups: Flavobacteriaceae (Elm/Kleiwiesen: 17.1/20.2%), Pseudomonadaceae (20/20.9%), Comamonadaceae (10.3/8.0%), Xanthomonadaceae (9.0/8.6%), Sphingobacteriaceae (8.1/9.5%), Enterobacteriaceae (2/1.6%) and Moraxellaceae (2.2/1.1%).

**Figure 3.**
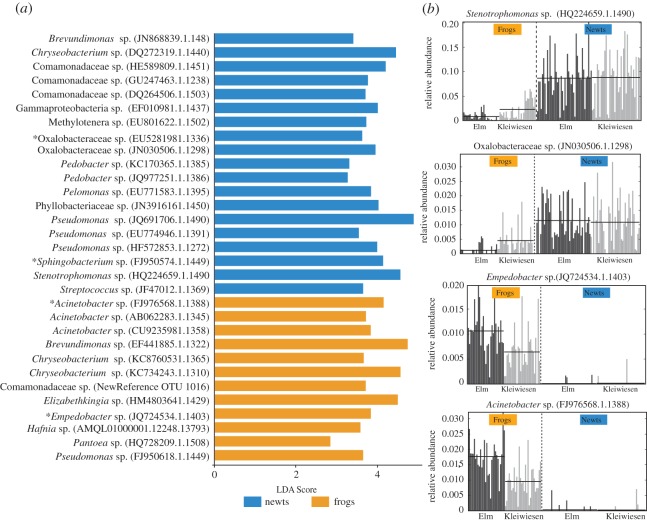
Bacterial OTUs that differed in relative abundance between frogs and newts across both Elm and Kleiwiesen. (*a*) LDA scores of detected OTUs from LEfSe analysis. Blue indicates OTUs that were differentially abundant on newts and orange indicates OTUs that were differentially abundant on frogs. Asterisks denote OTUs present in (*b*). (*b*) Relative abundances within the full community of four selected differential OTUs across samples from both locations. Two OTUs exhibit greater relative abundance in newts and two OTUs exhibit greater relative abundance in frogs. Each bar corresponds to an individual amphibian and the colours denote the sampling locations, Elm (black) and Kleiwiesen (grey) (both near Braunschweig, Germany). Electronic supplementary material, figures S1 and S2 provide relative abundance plots for the remaining 29 LEfSe-detected differentially abundant OTUs.

Of the 46 bacterial OTUs found on 100% of individuals of at least one amphibian species, LEfSe analysis revealed that 31 OTUs were differentially abundant between frog and newt skin communities across both sites. Nineteen OTUs were more abundant on newts and 12 OTUs were more abundant on frogs, and these differences were consistent across the two sampled locations (figure 3). In multiple cases, such as an *Empedobacter* sp. (frogs), *Acinetobacter* sp. (frogs), *Elizabethkingia* sp. (frogs) and a Phyllobacteriaceae sp. (newts), the LEfSe-detected OTUs were nearly absent from the communities of the other amphibian group; that is, for example, the *Empedobacter* OTU was significantly more abundant on frogs and absent from the communities of newts on all but four individuals that showed very low relative abundances (figure 3; electronic supplementary material, figures S1 and S2).

We defined core OTUs as those present on 90% of individuals of the respective species at a given location. The 90%-core microbiomes were as follows, for Elm and Kleiwiesen respectively: *R. dalmatina*—22 OTUs, *R. temporaria*—34 and 24 OTUs, *I. alpestris*—33 and 85 OTUs, *L. helveticus*—21 OTUs, *L. vulgaris*—32 and 14 OTUs, and *T. cristatus*—21 and 27 OTUs (table 3). Many of these core members were also found to be differentially abundant between frogs and newts with the LEfSe analysis (table 3). However, three OTUs were members of the core for all amphibian species across both sites, including a *Pseudomonas fluorescens*, an *Albidiferax* sp. and a *Chryseobacterium* sp. (Table 3).

**Table 3.** Core communities of frog and newt species at Kleiwiesen and Elm. Core is defined as OTUs present on at least 90% of individuals of the respective species at a given site. Greyed OTUs represent OTUs present in the core of all species, and asterisks indicate LEfSe-detected OTUs. Amphibian species are listed with the species sampled at both sites first, followed by those only sampled at one location. OTUs are ordered alphabetically within each bacterial phylum.

			Elm						Kleiwiesen			
phylum	taxa	SILVA ID	*Lissotriton vulgaris*	*Ichthyosaura alpestris*	*Triturus cristatus*	*Lissotriton helveticus*	*Rana temporaria*	*Rana dalmatina*	*Lissotriton vulgaris*	*Ichthyosaura alpestris*	*Triturus cristatus*	*Rana temporaria*
Proteobacteria	*Acinetobacter*	CU923598.1.1358*		X			X	X				X
	*Acinetobacter*	AB062283.1.1345*					X	X				X
	*Acinetobacter*	FJ774972.1.1224								X		
	*Acinetobacter*	GU356392.1.1407					X					
	*Acinetobacter calcoaceticus*	FJ976568.1.1388*					X	X				X
	*Albidiferax*	EF018476.1.1391	X	X	X	X	X	X	X	X	X	X
	*Bradyrhizobium*	KF408410.1.1205								X		
	*Brevundimonas*	EF441885.1.1322*					X					
	*Brevundimonas*	JN868839.1.1481*	X			X				X	X	
	Caulobacteraceae	HM438450.1.1432								X		
	*Cellvibrio*	JF135969.1.1355	X	X							X	
	*Cobetia*	FR744780.1.1252								X		
	Comamonadaceae	HE589809.1.1451*	X	X	X	X	X		X	X	X	X
	Comamonadaceae	DQ264506.1.1503*	X	X	X	X			X	X	X	
	Comamonadaceae	GU247463.1.1238*	X	X	X				X	X	X	X
	Comamonadaceae	AM157297.1.1226	X	X	X					X	X	
	Comamonadaceae	JQ965782.1.1392	X	X						X		
	Comamonadaceae	DenovoOTU1016*					X			X		
	Comamonadaceae	FJ802287.1.1223								X		
	*Comamonas*	HQ681963.1.1488					X					
	*Devosia*	JQ977237.1.1391								X		
	Enterobacteriaceae	FM163487.1.1535					X	X				
	Enterobacteriaceae	HM446001.1.1489								X		
	Gammaproteobacteria	EF010981.1.1437*	X	X		X				X	X	
	*Hafnia*	AMQL01000001.12248.13793*			X		X	X		X	X	X
	*Herbaspirillum*	KF188943.1.1289								X		
	*Iodobacter*	HM031078.1.1401								X		
	*Massilia*	HQ900361.1.1225								X		
	*Methylobacterium*	Z23159.1.1411								X		
	*Methylotenera*	EU801622.1.1502*	X		X					X		
	Oxalobacteraceae	EU528198.1.1336*	X	X	X	X				X	X	
	Oxalobacteraceae	JN030506.1.1298*	X	X	X				X	X		
	Oxalobacteraceae	DenovoOTU49								X		
	*Pantoea*	HQ728209.1.1508*	X	X		X	X		X		X	X
	*Pantoea*	FJ404760.1.1489					X	X				X
	*Pelomonas*	EU771583.1.1395*	X		X					X	X	
	*Peredibacter*	AB176231.1.1496								X		
	Phyllobacteriaceae	JN391616.1.1450*			X					X		
	*Pseudoalteromonas*	AM110980.1.1496								X		
	*Pseudomonas*	EU774946.1.1391*	X	X		X	X	X	X	X	X	X
	*Pseudomonas*	KF147070.1.1387	X	X		X	X	X		X	X	X
	*Pseudomonas*	HF572853.1.1272*	X	X	X	X			X	X	X	X
	*Pseudomonas*	FJ950618.1.1449*	X	X			X	X		X		X
	*Pseudomonas*	EU538168.1.1334								X		
	*Pseudomonas*	JQ926179.1.1490								X		
	*Pseudomonas*	JX221938.1.1390								X		
	*Pseudomonas fluorescens*	JQ691706.1.1490*	X	X	X	X	X	X	X	X	X	X
	*Psychrobacter*	DQ396354.1.1529								X		
	*Ralstonia*	JX222764.1.1493								X		
	Rhizobiaceae	DQ125688.1.1407	X	X					X	X		
	*Rhizobium*	EU704897.1.1316					X					
	*Rhodopseudomonas*	EU704957.1.1244								X		
	*Rhodopseudomonas*	GQ036328.1.1309								X		
	*Salinisphaera*	HM137558.1.1421								X		
	*Stenotrophomonas*	HQ224659.1.1422*	X	X	X	X	X	X		X	X	X
	*Stenotrophomonas*	GU553036.1.1504								X		
	*Stenotrophomonas*	JN411473.1.1371	X	X	X	X				X	X	
	*Sulfuricurvum*	AY510199.1.1245								X		
	*Undibacterium*	EU978820.1.1491		X								
	*Vogesella*	KC358338.1.1269								X		
Firmicutes	*Gemella*	JQ462192.1.1411								X		
	*Lactobacillus delbrueckii*	AF371475.1.1459								X		
	*Lactococcus lactis*	GQ267964.1.1475								X		
	*Streptococcus*	JF148823.1.1369								X		
	*Staphylococcus*	JF076235.1.1372	X	X			X			X		
	*Staphylococcus*	EF061902.1.1431								X		
	*Streptococcus salivarius*	JF047012.1.1369*		X	X					X		
Bacteroidetes	*Arcicella*	AB578880.1.1438								X		
	*Chryseobacterium*	JQ740255.1.1494	X	X			X	X		X		X
	*Chryseobacterium*	FR746066.1.1379						X				
	*Chryseobacterium*	KC734243.1.1310*	X	X	X	X	X	X	X	X	X	X
	*Chryseobacterium*	DQ272319.1.1440*	X	X	X	X	X	X		X	X	X
	*Chryseobacterium*	JQ977140.1.1396	X	X		X				X	X	X
	*Chryseobacterium*	KC876053.1.1365*					X	X				X
	*Chryseobacterium*	HQ259087.1.1391					X					
	*Chryseobacterium*	JQ977583.1.1412								X		
	*Elizabethkingia*	HM480364.1.1429*					X	X				
	*Empedobacter*	JQ724534.1.1403*					X	X				X
	*Ferruginibacter*	HM330982.1.1361								X		
	Flavobacteriaceae	DenovoOTU426					X			X		
	*Flavobacterium*	HM269115.1.1341								X		
	*Flavobacterium*	EF111092.1.1264								X		
	*Flavobacterium*	FQ859183.1179124.1180623	X	X	X	X	X		X	X	X	X
	*Flavobacterium*	HM278669.1.1346		X		X						
	*Flavobacterium*	AF493665.1.1275								X		
	*Flavobacterium*	DenovoOTU613								X		
	*Mucilaginibacter*	HQ327193.1.1485								X		
	*Pedobacter*	JQ977251.1.1386	X	X		X				X	X	
	*Pedobacter*	KC170365.1.1385*			X	X				X	X	
	*Pedobacter*	JQ977687.1.1435*								X		
	*Pedobacter* sp.	HM274322.1.1350	X	X		X				X	X	X
	*Sphingobacterium*	AY838484.1.1479					X	X				
	*Sphingobacterium faecium*	FJ950574.1.1449*	X	X	X		X		X	X	X	X
	*Sphingobacterium*	KC119153.1.1465	X	X			X	X	X	X		
	*Sphingobacterium*	AY856847.1.1449								X		
	*Wautersiella*	JX628858.1.1311					X					
Actinobacteria	*Arthrobacter*	AB696368.1.1333					X	X				
	*Brachybacterium*	AF041790.1.1436								X		
	*Brevibacterium*	FJ981663.1.1385								X		
	*Brevibacterium*	HE576064.1.1272								X		
	*Brevibacterium*	HQ418457.1.1490								X		
	*Leucobacter*	GQ358908.1.1484								X		
	Micrococcales	DenovoOTU1061								X		
	*Micrococcus*	FR682675.1.1488								X		
Planctomycetes	*Pirellula*	AJ290174.1.1324								X		
Verrucomicrobia	Verrucomicrobiaceae	GQ396806.1.1512								X		
	Verrucomicrobiaceae	DenovoOTU124									X	

## 4. Discussion

Cohabiting amphibian species, especially during their aquatic phase, offer the opportunity to test the effects of host factors in structuring skin microbial communities by removing extrinsic environmental factors. We hypothesized that amphibian species would harbour distinct bacterial communities, which was, in part, upheld by our results. When sampled at the same time-point, distinct communities were found to inhabit frog and newt hosts, but within these amphibian orders, the species did not have significantly different community structures. This pattern was consistent across both Elm and Kleiwiesen. We did not observe any phylogenetic patterns with respect to the microbiota of newt species (i.e. more closely related newt species did not exhibit greater similarity in their microbial communities). Our results suggest that frog and newt skins represent unique niches that promote growth of different bacterial taxa. While we cannot fully exclude differences in microhabitat preferences between frogs and newts as a factor influencing cutaneous bacterial composition, individuals of both groups are often found in the shallow edges of the ponds. The hosts probably have different mucosal components, including antimicrobial peptides, mucopolysaccharides, glycoproteins and toxins [35–37], that can influence community structure. Similar examples of host characteristics influencing the assembly of cutaneous microbiotas have been published from other animals; for example, in *Hydra*, antimicrobial peptides mediate the colonization of particular bacterial taxa on the epithelial cells [38], and in humans, microbial community structure is influenced by skin pH and moisture [39]. The newt species in the present study belong to genera that are all known to contain tetrodotoxins [40], and at least *T. cristatus* also contains steroidal alkaloids [41]. Such alkaloids have been found to have antimicrobial properties [18] and could contribute to shaping the microbiota on these newts. On the other hand, *Rana temporaria* is known to produce antimicrobial peptides [42–44] which are not known from salamandrid newts. It is striking that the host effect is so strong that several bacterial OTUs are regularly found on all individuals and species of either frogs or newts, across the two localities, but are almost absent from individuals of the other amphibian group, respectively (figure 3). Newts appeared to exhibit stronger filtering: three bacterial OTUs (an *Empedobacter* sp.*,* an *Acinetobacter* sp. and an *Elizabethkingia* sp.) that were abundant on frog skin were absent in nearly all sampled newts. Frogs, on the other hand, had a less drastic pattern; most OTUs exhibiting greater relative abundance on newts were still regularly present on frog, albeit in low relative abundances.

All 31 differentially abundant OTUs were also detected in samples taken from the pond water and matched to bacteria from environmental samples of water or soil in BLAST searches, suggesting that environmental transmission is a major factor in shaping and maintaining amphibian cutaneous microbial communities [5].

The observed pattern in this study also mirrors other amphibian studies where amphibians from different genera or orders have been found to have different bacterial communities [6,10–13,45]. In addition, the common bacterial groups found here on European amphibian hosts (i.e. Pseudomonadaceae, Moraxellaceae, Xanthomonadaceae, Comamonadaceae, Enterobacteriaceae) were strikingly similar to the bacteria found on other amphibians globally [10–13], further suggesting that the properties of amphibian skin may act as selective forces favouring particular bacterial taxa.

Amidst the drastic differences between frogs and newts in the system studied herein, some common core OTUs were detected across all sampled amphibian species, including *Pseudomonas* spp. and *Chryseobacterium* spp., which are known to have fungal inhibiting properties [2,46–48] and could possibly contribute to their cutaneous defence against *Bd*. This fungal pathogen is known to be present within these ponds [49]; therefore, such bacteria may play a role in protection against *Bd*. However, since the pathogen occurs at low prevalences of up to 6% across species, it is unlikely that microbial differences among species herein were caused by *Bd* infection. The apparent occurrence of bacteria present on amphibians also in the environment may suggest that they are not *a priori* specialized. However, it remains to be tested whether specific strains, indistinguishable in the short 16S sequence used for Illumina-based amplicon studies, might have specific components within their flexible genomes that make them better colonizers of the amphibian skin. In future studies, it will be of particular interest to test if the frog versus newt strains, while identical by 16S, might have specific genomic or metabolic differences favouring colonization, e.g. by conferring resistance to the host-specific antimicrobial defence mechanisms.

## Supplementary Material

Supplementary Figure 1: LEfSe-identified OTUs with greater abundance on newts

## Supplementary Material

Supplementary Figure 2: LEfSe-identified OTUs with greater abundance on frogs

## Data accessibility

All sequence data have been deposited in the SRA Database under SRP074714 (Bioproject PRJNA320969).
