# Supplementary material for: Skin microbiota differs drastically between co-occurring frogs and newts
Source: R Soc Open Sci. 2017 Apr 5;4(4):170107. doi: 10.1098/rsos.170107 (PMC5414276; doi:10.1098/rsos.170107)
Supplement: Supplementary Figure 1: LEfSe-identified OTUs with greater abundance on newts [file rsos170107supp1.pdf]

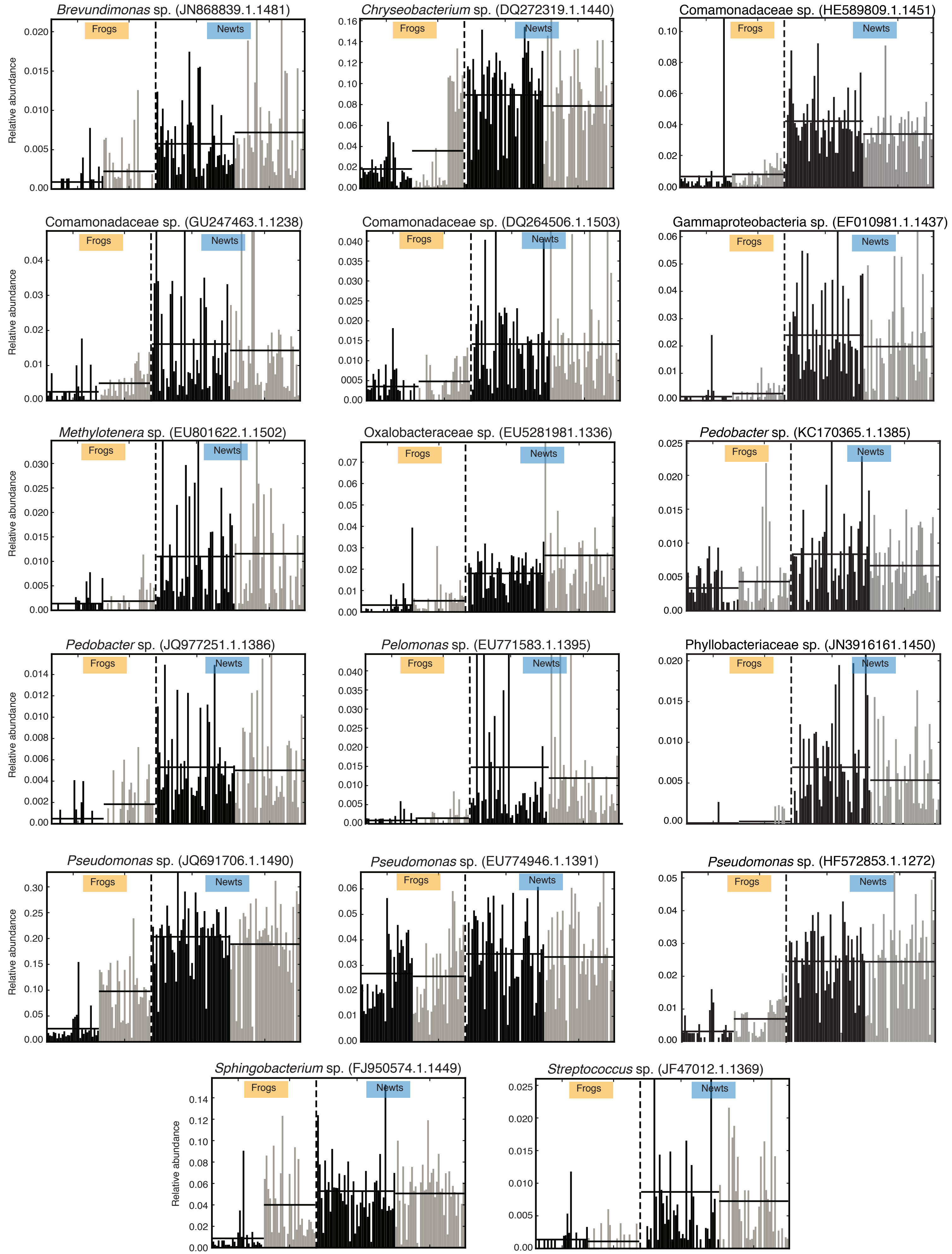

Supplementary Figure 1: Relative abundance patterns of LEfSe identified OTUs with greater abundance on newts. relative abundance plots for the additional seventeen OTUs detected by LEfSe analysis to be differentially more abundant on newts at each sampled location (Elm = black and Kleiwiesen = gray).
