# Supplementary material for: Skin microbiota differs drastically between co-occurring frogs and newts
Source: R Soc Open Sci. 2017 Apr 5;4(4):170107. doi: 10.1098/rsos.170107 (PMC5414276; doi:10.1098/rsos.170107)
Supplement: Supplementary Figure 2: LEfSe-identified OTUs with greater abundance on frogs [file rsos170107supp2.pdf]

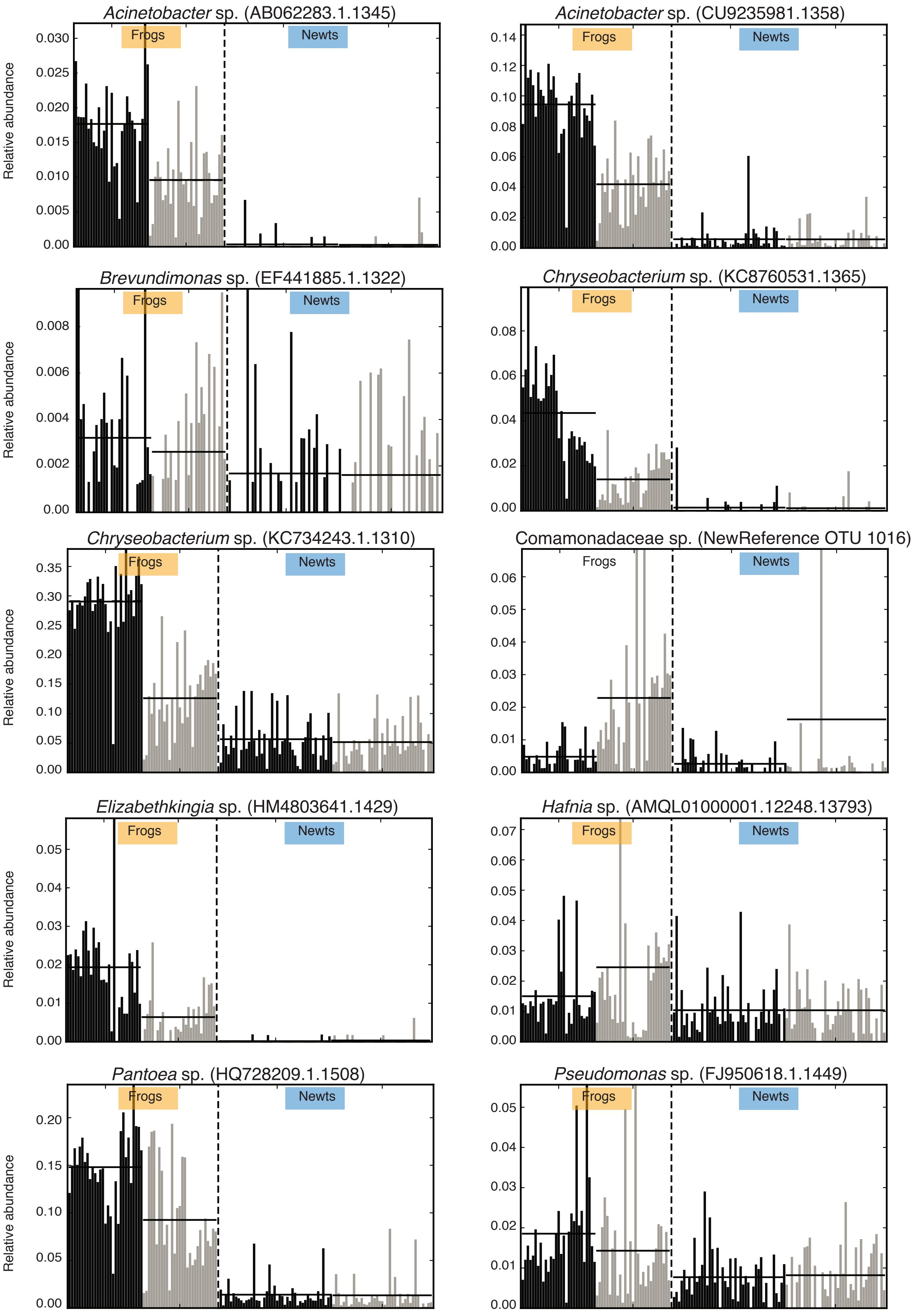

Supplementary Figure 2: Relative abundance patterns of LEfSe identified OTUs with greater abundance on frogs. Relative abundance plots for the additional ten OTUs detected by LEfSe analysis to be differentially more abundant on frogs at each sampled location (Elm = black and Kleiwiesen = gray).
